# Refractory pituitary adenoma: a novel classification for pituitary tumors

**DOI:** 10.18632/oncotarget.13274

**Published:** 2016-11-10

**Authors:** Congxin Dai, Ming Feng, Xiaohai Liu, Sihai Ma, Bowen Sun, Xinjie Bao, Yong Yao, Kan Deng, Yu Wang, Bing Xing, Wei Lian, Dingrong Zhong, Wenbin Ma, Renzhi Wang

**Affiliations:** ^1^ Department of Neurosurgery, Peking Union Medical College Hospital, Chinese Academy of Medical Sciences and Peking Union Medical College, Beijing 100730, China; ^2^ Department of Pathology, Peking Union Medical College Hospital, Chinese Academy of Medical Sciences and Peking Union Medical College, Beijing 100730, China

**Keywords:** pituitary adenomas, refractory, resistance, recurrence, aggressive

## Abstract

Pituitary adenomas are classified as typical or atypical, invasive or noninvasive, and aggressive or nonaggressive based on pathological features, radiological findings, and clinical behavior. Only pituitary tumors with cerebrospinal and/or systemic metastasis are considered malignant carcinomas. However, some pituitary adenomas with high Ki-67 indexes exhibit aggressive behaviors, such as rapid growth, early and frequent recurrence, and resistance to conventional treatment, even in the absence of metastasis. Novel terminology is needed to define these tumors. Here, we propose the use of the term “refractory pituitary adenoma” to define malignant pituitary tumors exhibiting 1) a high Ki-67 index and rapid growth, 2) early and high frequency of recurrence, 3) resistance to conventional treatments and/or salvage treatment with temozolomide (TMZ), 4) poor prognosis, 5) and a lack of cerebrospinal or systemic metastases. To illustrate the utility of this refractory pituitary adenoma classification and the difficulty in managing disease in these patients, we examined twelve clinical cases. Correctly identifying refractory pituitary adenomas is crucial for improving patient prognoses. Early identification might encourage the early use of aggressive therapeutic strategies to prevent or delay recurrence.

## INTRODUCTION

Pituitary adenomas arise from adenohypophyseal cells and account for 10–15% of all intracranial neoplasms, which are the second most common type of intracranial tumor [1]. Although most pituitary tumors are noninvasive, exhibiting slow expansive growth and remaining within the sella or displacing the surrounding tissues, up to 25–55% of pituitary adenomas are invasive and actively infiltrate adjacent tissues, such as the cavernous sinuses, bone, sphenoid sinuses, and, less commonly, blood vessels and nerve sheaths [2, 3]. In 2004, the World Health Organization (WHO) classification categorized pituitary adenomas as either typical, atypical, or carcinoma. Atypical adenoma is characterized by a Ki-67 labeling index greater than 3%, extensive p53 immunoreactivity, and an elevated mitotic index [4]. According to the current classification, only pituitary tumors that involve systemic metastasis, including one or several cerebral or meningeal metastases, are considered truly malignant; such carcinomas are very rare and only account for 0.2 % of all pituitary tumors [5]. “Aggressive” adenomas, which are intermediate in phenotype between typical pituitary adenomas and pituitary carcinomas, exhibit distinct clinical characteristics, frequently recur, and are often resistant to conventional treatments [6]. Although pituitary adenomas can be classified as invasive or noninvasive, typical or atypical, and aggressive or nonaggressive, these categorizations do not accurately describe malignant pituitary tumors without cerebrospinal and/or systemic metastasis that grow rapidly, have high Ki-67 indexes, recur frequently and early, are resistant to conventional treatments and/or salvage treatment with TMZ, and ultimately result in death.

Furthermore, the typical/atypical WHO classification for adenomas does not always correlate with clinical behavior or radiological features; typical pituitary adenomas may be invasive and have aggressive phenotypes [7], while many atypical pituitary adenomas are noninvasive and nonaggressive [8]. Invasive pituitary adenomas also have a variety of different pathological features and clinical courses. Some “invasive” pituitary adenomas display typical adenoma pathology and benign behavior, even when invasion of the dura, bone, and/or the surrounding anatomical structures has occurred [9]. Additionally, although the term “aggressive” has been used to describe pituitary adenomas with rapid growth and recurrence and resistance to conventional treatments, “aggressive” and “invasive” are interpreted differently by different clinicians; these terms are often used interchangeably, and an accurate definition of, and diagnostic criteria for aggressive primary tumors are needed [10, 11]. For these reasons, the current pituitary tumor classification system may require updating, and malignant pituitary tumors without systemic metastases in particular need to be properly defined.

Here, we propose the use of the term “refractory pituitary adenoma” to define pituitary adenomas with a high Ki-67 index, rapid growth, frequent recurrence, and resistance to conventional treatments and/or TMZ. The criteria for diagnosing refractory pituitary adenomas are as follows: 1) tumor infiltrates adjacent structures according to radiological results or intraoperative findings; 2) tumor Ki-67 index is greater than 3% and growth velocity is more than 2% monthly; 3) current treatments fail to control tumor growth and/or hormonal hypersecretion; 4) tumor recurrence occurs within 6 months after surgery. Here, we examined twelve refractory pituitary adenoma cases to support this disease classification and to illustrate the difficulties involved in diagnosing and treating these tumors.

## PRESENTATION OF SELECTED CASES

### Case one

**Figure 1:**
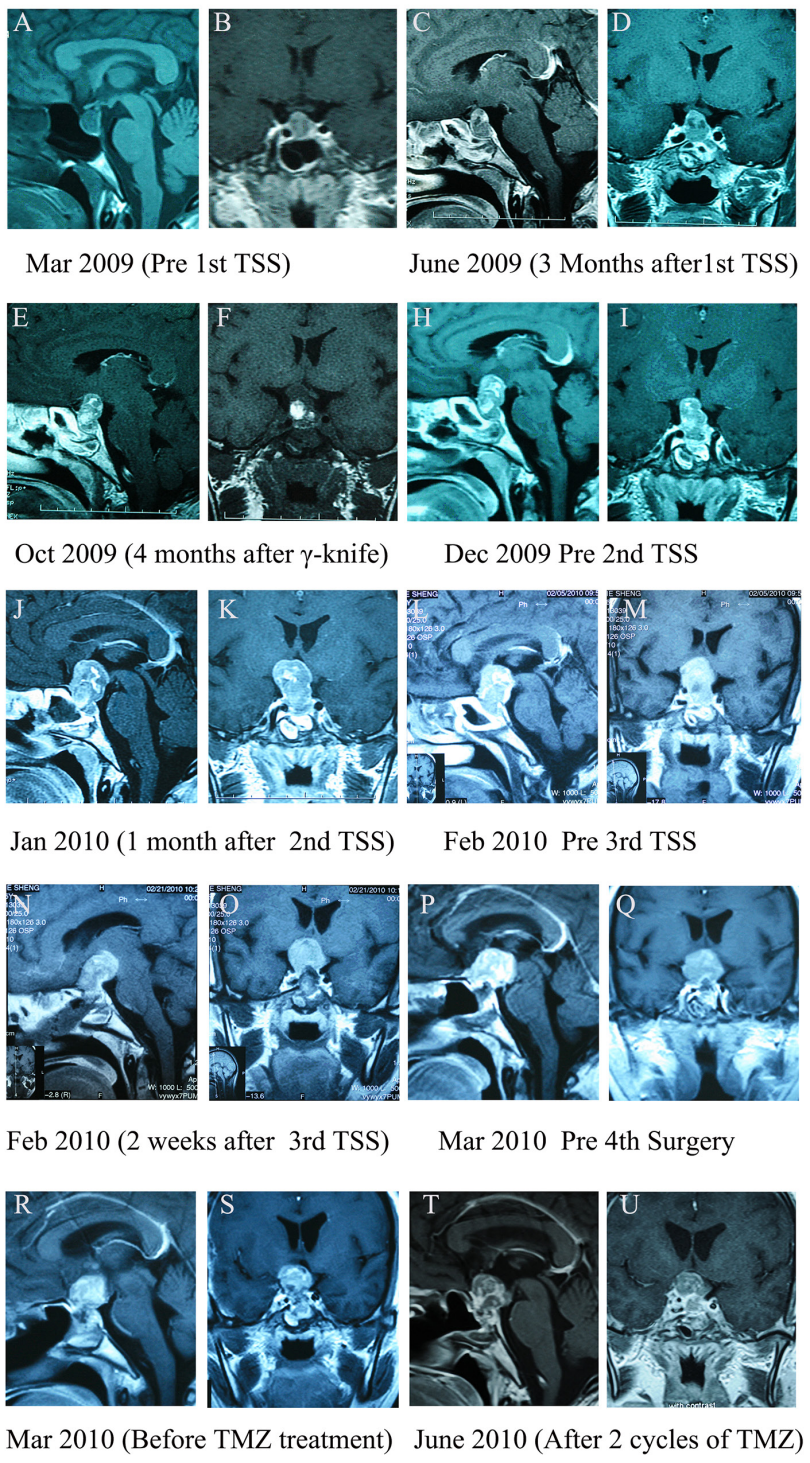
Pituitary MRI images for the case one patient **A** and **B.** Pre-operative T1 weighted images. **C** and **D.** 3 months after the first transsphenoidal surgery (TSS). **E** and **F.** 4 months after gamma knife surgery (GKS). **H** and **I.** 6 months after GKS. **J** and **K.** 1 month after the second TSS. **L** and **M.** 2 months after the second TSS. **N** and **O.** 2 weeks after the third surgery. **P** and **Q.** 1 month after the third surgery. **R** and **S.** 2 weeks after the fourth operation, before TMZ treatment. **T** and **U.** After two cycles of TMZ therapy.

**Figure 2:**
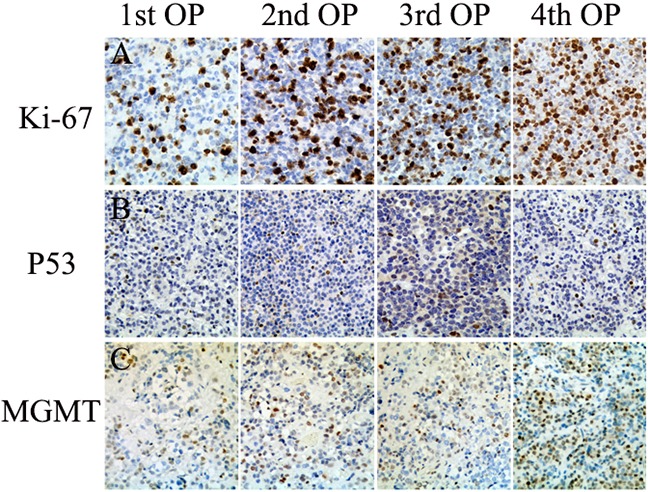
Histopathological findings in resected pituitary tumors from the case one patient **A.** Immunohistochemistry for Ki-67 nuclear labeling demonstrating that the Ki-67 index increased as operation frequency increased (20%, 30%, 40%, and 50%) (20×). **B.** Strong nuclear staining for p53 was observed in some tumor cells(20×). **C.** O6 methylguanine-DNA methyltransferase (MGMT) immunohistochemistry demonstrating that the proportion of MGMT-positive tumor cells in resected tissue increased as operation frequency increased (20%, 25%, 30%, 50%)(20×).

A 46-year-old female patient presented in March 2009 with headaches and visual impairment; a macroadenoma (12 × 15 × 13 mm) with an invasion of the suprasellar cistern was detected by MRI (Figure 1A and 1B), and laboratory test results were normal. The patient was initially diagnosed with non-functional pituitary adenoma (NFPA) and underwent an initial transsphenoidal surgery (TSS) in March 2009. Because the tumor was very firm and fibrous, it was only partially removed, which improved the patient's headaches, but not her visual impairment. Pathological testing of the initial tumor tissue indicated a high Ki-67 index (20%), p53-positive immunostaining in some tumor cells, and MGMT-positive immunostaining in 20% of tumor cells (Figure 2). In June 2009, gamma knife surgery (GKS) treatment was performed to remove the residual tumor. However, MRI 4 months after GKS treatment revealed that the tumor continued to grow (21 × 25 × 18 mm) (Figure 1E and 1F). In December 2009, the patient experienced more severe visual impairment and headache accompanied by nausea and vomiting, and MRI revealed significant enlargement of the residual tumor (19 × 25 × 22 mm) (Figure 1H and 1I). In December 2009, a second TSS was performed and the tumor was partially resected. Histology revealed that the Ki-67 index increased to 30%, and the percentage of MGMT-positive cells increased slightly as well (Figure 2). Despite the second operation, the patient's visual impairment became more severe, and the visual field defect worsened in January 2010. Significant tumor regrowth with compression of the optic chiasm and invasion into the third ventricle was observed by MRI (Figure 1J and 1K). The patient was then referred to our hospital and a third TSS was performed. The tumor was again only partially resected because it was extremely firm and fibrous. Pathological tests revealed that the Ki-67 index increased to 40% and the MGMT-positive cell percentage increased to 30% (Figure 2). To exclude pituitary carcinoma, a Positron-Emission Tomography (PET) scan was performed; there was no evidence of metastasis. In March 2010, a fourth operation was performed to partially resect the tumor using a right frontotemporal approach due to rapid regrowth. Pathological tests indicated that mitotic activity increased, Ki-67 index increased to 50%, and MGMT-positive cell percentage increased to 50% (Figure 2). In May 2010, the patient received TMZ treatment under the standard regimen of 200 mg/m^2^/d for 5 days of a 28-day cycle. The size of the tumor did not change after 2 cycles of TMZ treatment, although suspensive necrosis did change (Figure 1T and 1U). However, TMZ treatment was discontinued at the patient's request, and she died of inflammation in October 2010.

### Case two

**Figure 3:**
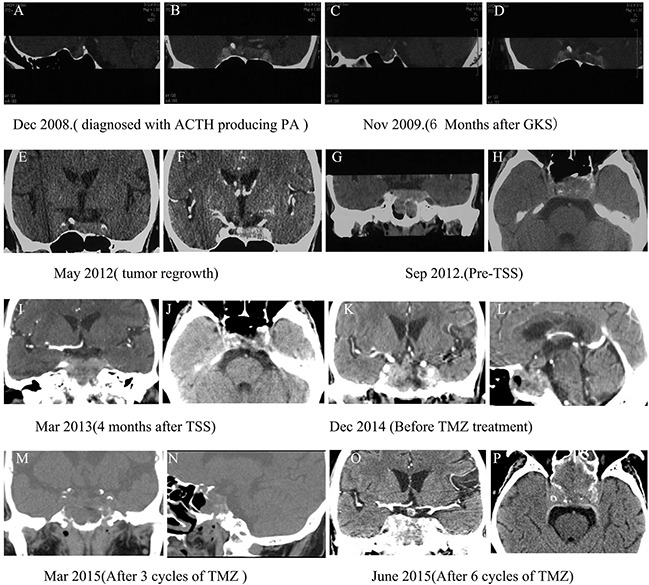
Computed Tomographic (CT) images of pituitary tumors in the case two patient **A** and **B.** CT images at initial diagnosis. **C** and **D.** Six months after GKS. **E** and **F.** CT scan from May 2012 showing significant tumor progression. **G** and **H.** CT scans from September 2012 showing significantly enlarged pituitary masses that invaded the sphenoid and cavernous sinuses. **I** and **J.** CT scan 4 months after TSS showing residual tumor regrowth. **K** and **L.** Before TMZ administration. **M** and **N.** After 3 cycles of TMZ. **O** and **P.** After 6 cycles of TMZ.

**Figure 4:**
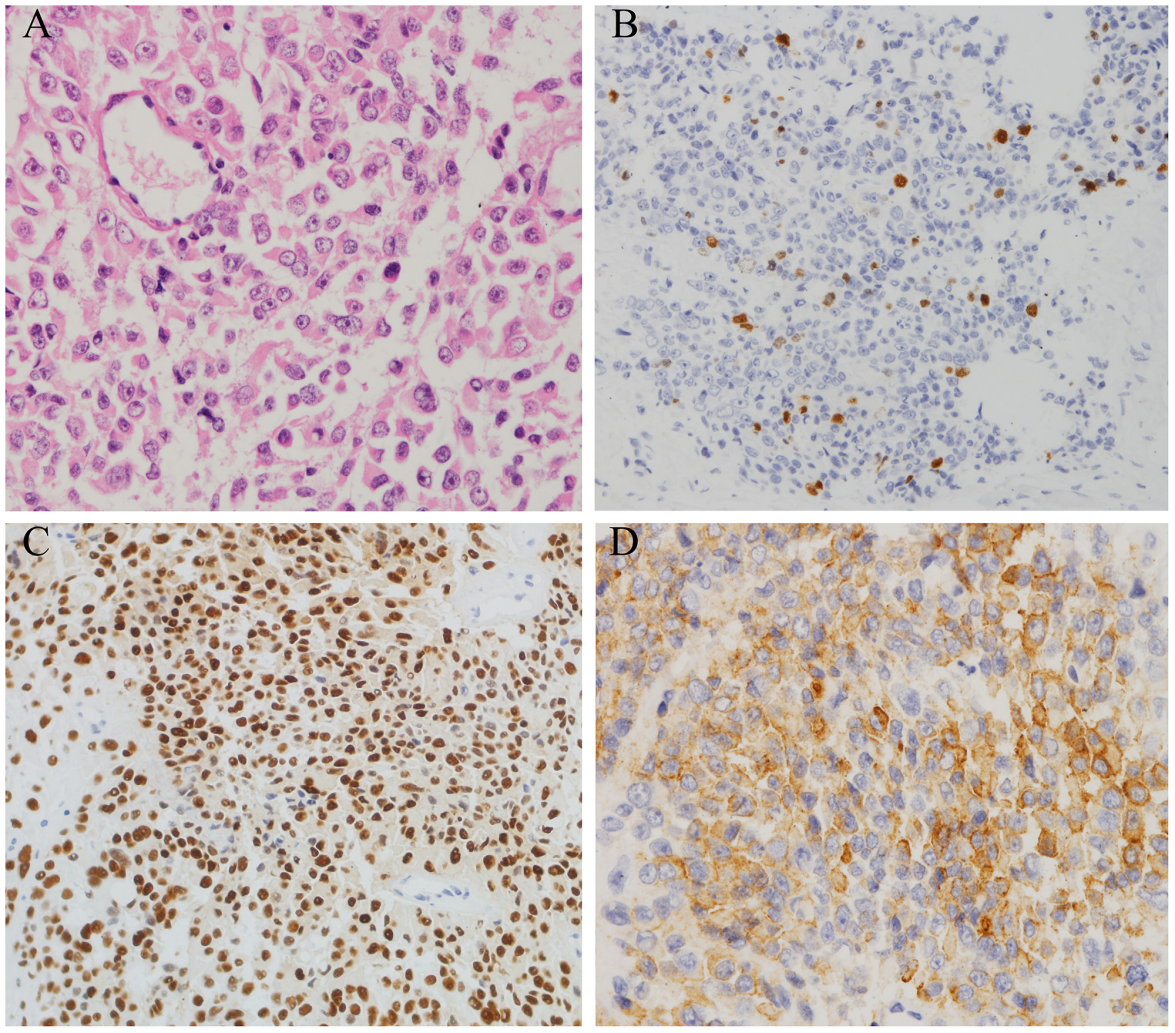
Histopathological findings in resected pituitary tumors from the case two patient **A.** Haematoxylin and eosin (H&E) staining of pituitary tumor (40×); **B.** The Ki-67 index was greater than 5% (40×); **C.** Diffuse and strong nuclear p53 staining (arrow) was observed in 90% of tumor cells (40×); **D.** 90% of tumor cells were positive for ACTH (40×).

**Figure 5:**
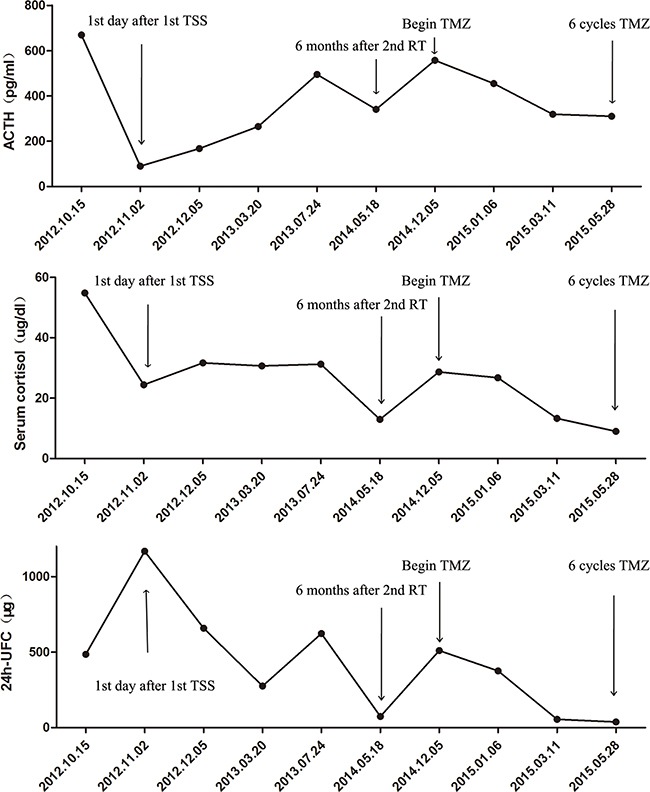
Graph depicting changes in ACTH, serum cortisol, and 24h-UFC (urine free cortisol) levels after various treatments and following treatment with temozolomide (TMZ) in the case two patient TSS: transsphenoidal surgery; RT: radiotherapy.

A 64-year-old male was referred to our hospital in 2009 due to a 1-year history of facial swelling and weight gain. CT scans revealed a pituitary mass (2.3 × 1.3 × 1.1 cm) involving the cavernous sinuses (Figure 3A and 3B); laboratory test results were within normal limits with the exception of ACTH levels, which were elevated to 113 pg/mL (normal <46 pg/mL). The patient did not consent to surgery because he had undergone a permanent pacemaker implantation for sinus bradycardia in 2008 and was concerned about the risks associated with anesthesia and operation. Therefore, GKS was performed instead of surgery in May 2009. Six months after GKS, the patient's face puffiness and weight gain symptoms had improved, and CT scans showed that the pituitary mass decreased slightly in size (Figure 3C and 3D). Although the patient also developed secondary hypothyroidism, the associated symptoms improved after levothyroxine replacement treatment. In June 2011, the patient's face puffiness and weight gain returned, and CT scans in May 2012 showed significant progression of the tumor (Figure 3E and 3F). In August 2012, the patient also began to experience visual impairment in the left eye. CT scans revealed that the pituitary was greatly enlarged and that the sphenoid and cavernous sinuses were involved (Figure 3G and 3H). Laboratory tests in October 2012 showed that ACTH, serum cortisol, and 24h-UFC levels were elevated to 669 pg/mL, 54.84 μg/dL (normal: 4-22.3 μg/dL), and 485 μg (normal: 12.3-103 μg), respectively (Figure 5). In November 2012, TSS was performed and the tumor was partially resected. Pathology revealed ACTH-positive immunostaining in 80% of cells, a Ki-67 labeling index (LI) of greater than 5%, and strong immunoreactivity for p53 in nearly 90% of the tumor cells (Figure 4). After the operation, ACTH and serum cortisol levels decreased to 90.1 pg/mL and 24.43 μg/dL, respectively; however, 24h-UFC levels increased to 1168 μg (Figure 5). In May 2013, the patient presented with further visual impairment and diplopia in left eye and moon face. CT scans indicated that the residual tumor had regrown (Figure 3H and 3I). The patient's ACTH and serum cortisol levels increased to 495 pg/mL and 31.25 μg/dL respectively, while his 24h-UFC levels decreased to 623 μg (Figure 5). In August 2013, radiotherapy was performed to treat further tumor progression. Six months after radiotherapy, ACTH, serum cortisol, and 24h-UFC levels decreased to 341.0 pg/mL, 12.95 μg/dL, and 73.60 μg, respectively (Figure 5), and the patient's Cushing syndrome, but not his visual impairment and diplopia, improved significantly. However, in December 2014, the patient presented with an aggravation of the visual impairment in the left eye and the right abducent nerve palsy, and CT scan revealed that the pituitary adenoma had regrown and infiltrated through the sphenoid bones into the sphenoid and cavernous sinuses (Figure 3J and 3K). The patient's ACTH, serum cortisol, and 24h-UFC levels had increased to 557 pg/mL, 28.68 μg/dL, and 510 μg, respectively (Figure 5). After obtaining written consent, TMZ was administered at 200 mg/m^2^ daily for 5 days every 28 days beginning in December 2014. After 3 cycles of TMZ, no virtual alteration in tumor size was observed by CT scan (Figure 3L and 3M). However, ACTH, serum cortisol, and 24h-UFC levels decreased to 319 pg/mL, 13.3 μg/dL, and 55.29 μg, respectively (Figure 5), and the patient's clinical symptoms improved significantly, including the recovery of extraocular movements and a reduced hypertension. After six cycles of TMZ treatment, serum cortisol and 24h-UFC levels had fallen into the normal range, and the ACTH level decreased to 310 pg/mL (Figure 5). However, the patient presented with blepharoptosis in the right eye, hoarseness, bucking, and dysphagia, and the CT scan indicated that the tumor had enlarged rapidly and infiltrated the sphenoid and cavernous sinuses (Figure 3). The patient then declined to continue TMZ treatment and returned home for terminal care.

### Case three

**Figure 6:**
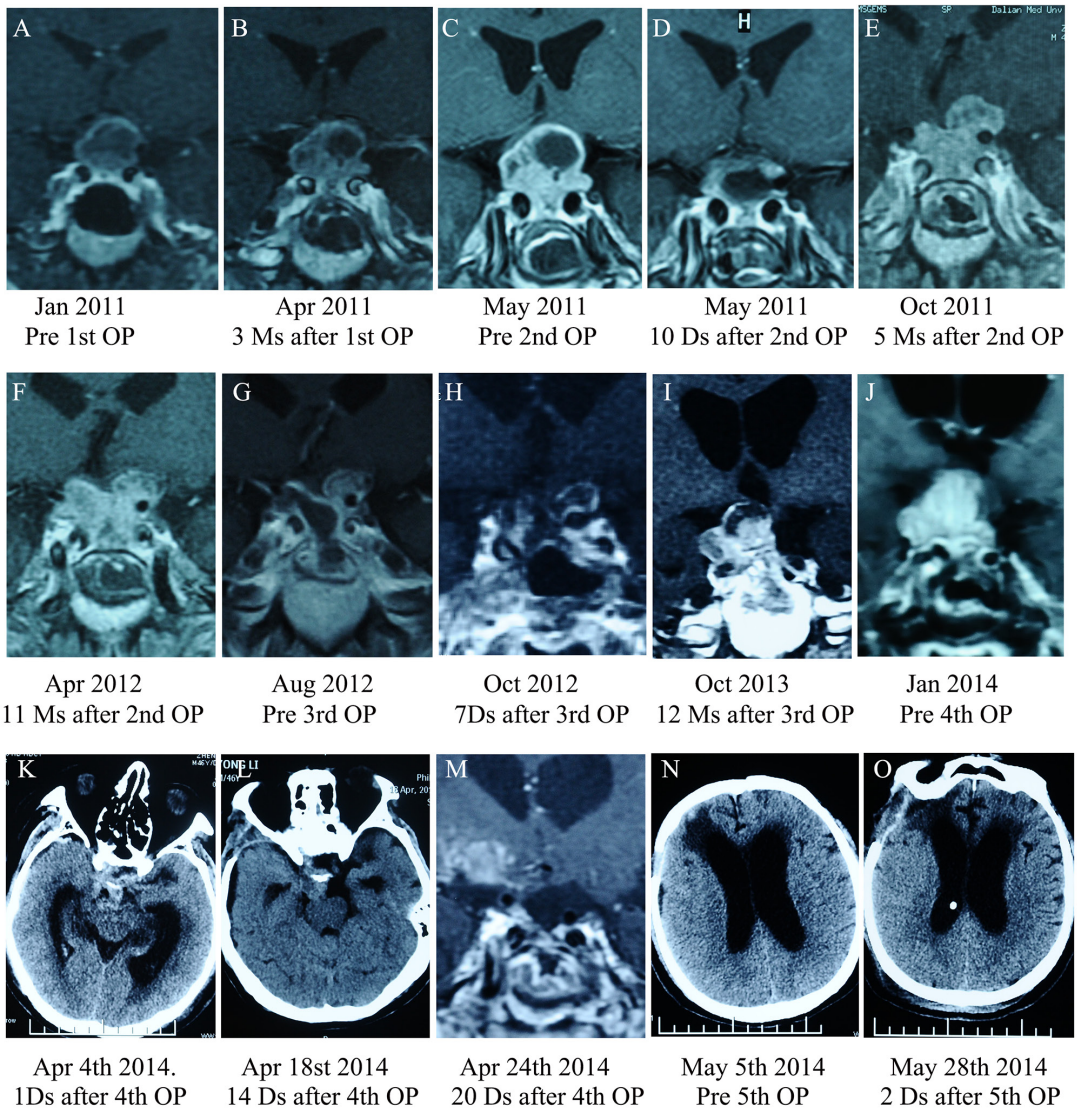
Pituitary MRI images for the case three patient **A.** MRI images at initial diagnosis. **B.** 3 months after the first operation. **C.** Pre-operative MRI image prior to the second TSS. **D.** 10 days after the second operation. **E.** 5 months after the second operation. **F.** 11 months after the second operation. **G.** Before the third operation. **H.** 7 days after the third operation. **I.** 1 year after the third operation. **J.** Before the fourth operation. **K, L and M.** CT scan after the fourth operation showing subtotal tumor removal. **N.** In May 2014, the patient presented with hydrocephalus, and a ventriculoperitoneal shunt was implanted; hydrocephalus subsequently improved **O.**

**Figure 7:**
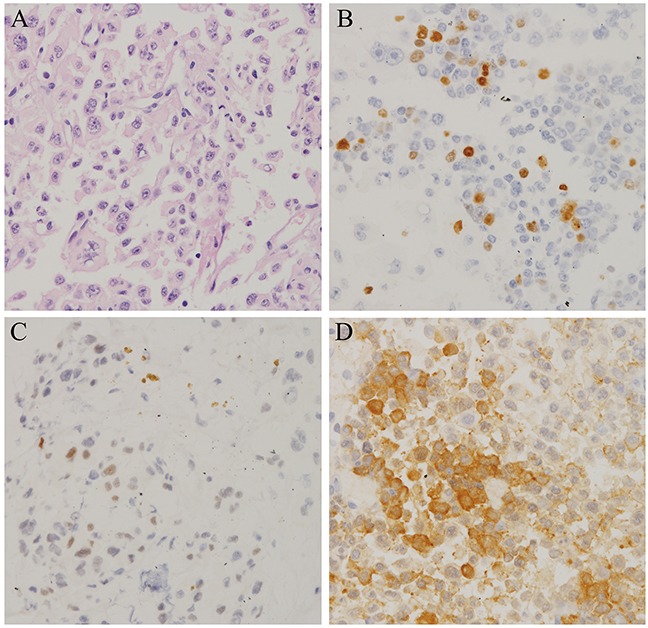
Histopathological findings in resected pituitary tumors from the case three patient **A.** Haematoxylin and eosin (H&E) staining of pituitary adenomas(40×). **B.** The Ki-67 index was greater than 10% (40×). **C.** Immunohistochemistry for p53 showing very few immunopositive cells (3 %) (40×). **D.** The proportion of ACTH-positive tumor cells in resected tissue increased as operation frequency increased (40×).

A 46-year-old male presented in January 2011 with visual impairment in the right eye and without endocrine-related symptoms. MRI revealed a macroadenoma (23 × 21 × 16 mm) that had invaded the right cavernous sinus (Figure 6A). TSS was performed in January 2011 with a subtotal resection of the tumor, leading to visual improvement. Pathological test results indicated that the Ki-67 index was greater than 3%. In April 2011, the patient again experienced hemianopsia in the right eye, and MRI scan showed that the tumor had recurred with infiltration of the cavernous sinus (22 × 20 × 17 mm) (Figure 6B). A second surgical intervention was performed to partially resect the tumor using a transcranial approach (Figure 6D), resulting in visual improvement. Pathological tests revealed that the Ki-67 index was greater than 10% (Figure 7). However, five months after the second surgery, MRI revealed regrowth of the residual tumor with invasion of the suprasellar cistern and left cavernous sinuses (Figure 6E). In January 2012, the patient presented with typical Cushing syndrome, and his ACTH and serum cortisol levels had increased to 119 pg/mL and 49.5 μg/dL, respectively. Tumor recurrence (30 × 25 × 23 mm) was detected by MRI scans in April 2012 (Figure 6F). In October 2012, a third TSS was performed and the tumor was partially resected (Figure 6H). Pathology revealed strong ACTH-positive immunostaining in 90% of the cells and a Ki-67 labeling index (LI) of greater than 10%, and weak immunoreactivity for p53 was observed in almost 5% of tumor cells (Figure 7). Although the patient underwent fractionated stereotactic irradiation of the residual tumor in December 2012, he began to experience headaches, visual impairment in both eyes, and blepharoptosis in the right eye, and MRI scans in January 2014 revealed that rapid tumor regrowth had occurred with infiltration of the right cavernous sinus (30 × 25 × 27 mm) (Figure 6J). A fourth subtotal tumor resection surgery was performed in April 2014 using a transcranial approach (Figure 6M), and resulted a transient improvement in headache and visual impairment symptoms. However, the patient presented with hydrocephalus in May 2014 (Figure 6N); a ventriculoperitoneal shunt was implanted, which initially improved his hydrocephalus (Figure 6O). However, the tumor continued to grow rapidly, and the patient died of severe hydrocephalus in October 2014.

The clinical characteristics of the remaining refractory pituitary adenomas examined here are presented in Table 1. Growth rates were determined by calculating the velocity of tumor volume increases using a stereological method based on the Cavalieri principle [12].

**Table 1:** Clinical feature of refractory pituitary adenomas

Case	Sex	age (y)	PA	Invasive (Y or N)	Ki-67 (%)	growth rate(%/m)	Atypical (Y or N)	Surgery (n)	RT	TMZ (cycles)	Outcome
1	F	46	NFPA	Y	40%	2.7%	Y	3	GKS	2	Dead
2	M	64	ACTH	Y	5%	3.3%	Y	1	GKS	6	Dead
3	M	46	ACTH	Y	>10%	2.6%	Y	4	Radiation	N	Dead
4	M	75	NFPA	Y	>3%	4.2%	Y	1	N	5	Progression
5	F	43	ACTH	Y	>3%	3.1%	N	1	Radiation	6	Progression
6	F	29	GH	Y	3%	2.3%	N	2	GKS	2	Progression
7	M	50	NFPA	Y	40%	2.8%	Y	3	GKS	3	Progression
8	F	30	NFPA	Y	>3%	2.6%	N	1	N	5	Progression
9	M	28	NFPA	Y	3%	2.9%	Y	4	Radiation	2	Progression
10	F	55	PRL	Y	10%	3.3%	N	1	GKS	6	Progression
11	F	66	NFPA	Y	5%	4.6%	Y	2	N	6	Dead
12	M	30	NFPA	Y	>3%	3.7%	Y	2	N	2	Progression

GKS: gamma knife surgery; RT: radiotherapy; TMZ: temozolomide; N: no

## DISCUSSION

These cases illustrate the complexity of classifying and treating malignant pituitary adenomas that are not considered pituitary carcinomas due to the absence of systemic or craniospinal metastasis. The pituitary tumors examined here had particularly aggressive clinical courses, progressed rapidly, and ultimately resulted in death, despite multiple surgeries, radiotherapy, and/or salvage treatment with TMZ. In addition, these tumors also had very high Ki-67 proliferative indexes, which is a common histological feature of pituitary carcinoma; this further supports that the tumors examined here were malignant in nature. However, according to current classification conventions, these tumors are considered benign pituitary adenomas due to the absence of cerebrospinal or systemic metastases. There is presently no reasonable term to adequately define this subtype of pituitary tumor, which is not sufficiently described by the typical or atypical, invasive or noninvasive, and aggressive or nonaggressive adenoma classifications.

Furthermore, the relationships between these “aggressive,” “invasive,” and “atypical” classifications are complex. In fact, the aggressive nature of some pituitary adenomas with local invasiveness is frequently undetected due to relatively benign histopathological results. Additionally, typical morphological indicators, such as high cellular mitotic activity, pleomorphism, and nuclear atypia, correlate poorly with the malignant potential of pituitary adenomas [13]. Furthermore, radiological findings do not always correlate with pathological findings or clinical behavior [14]. For example, some invasive pituitary adenomas are relatively benign, typical adenomas (Ki-67<3%) without any aggressive clinical behavior, while other invasive pituitary adenomas are atypical adenomas (Ki-67>3%) with aggressive clinical behaviors, high rates of recurrence, and resistance to conventional treatments and/or TMZ [15]. Atypical adenomas can be either invasive or noninvasive; similarly, not all typical adenomas have aggressive clinical behavior [8]. In addition, not all aggressive adenomas show the pathological features that characterize atypical adenomas, and radiological tests may indicate that they are noninvasive in early stages [11]. Thus, the terms “invasive,” “atypical,” and “aggressive” should only be used to describe radiological and surgical findings, pathological features, and clinical behavior, and cannot comprehensively describe the malignant features of these pituitary adenomas.

Here, we propose the use of the term “refractory pituitary adenoma” to define pituitary tumors which exhibit a distinctive disease course compared to benign adenomas and pituitary carcinomas (Supplementary Table S1). This terminology may more accurately reflect the malignant features and aggressive nature of these pituitary adenomas, which lack systemically metastases. Here, we describe the radiological, pathological, and clinical characteristics of these refractory pituitary adenomas, which are aggressive-invasive adenomas with a Ki-67 LI greater than 3%. This new classification may help overcome critical limitations in the characterization of pituitary tumors.

Aggressive pituitary adenomas, like refractory pituitary adenomas, are characterized by earlier and more frequent recurrences and resistance to conventional treatments. However, the term “aggressive” is poorly-defined, lacking clear diagnostic criteria and classification systems, and different clinicians use the term differently [16]. Furthermore, there are no specific biomarkers for conclusively identifying aggressive pituitary adenomas in the clinical setting [17]. Proliferation of the Ki-67 antigen, which is a major indicator for distinguishing typical from atypical adenomas, may help to predict the aggressive potential of pituitary adenomas. A Ki-67 index greater than 3% identifies adenomas as invasive as opposed to noninvasive [18]; although some studies report that a Ki-67 index greater 10% may also be indicative of increased pituitary adenoma aggressiveness [19], inconsistent results among studies limit the utility of this measure [20–22]. In contrast, clinically aggressive pituitary adenomas can be typical or atypical, and not all aggressive pituitary adenomas are associated with a high Ki-67 index [23, 24]. Positive p53 immunostaining, another criterion for atypical adenoma according to the WHO classification, may also have a diagnostic application as a marker for aggressive behavior [25]. Previous studies have found that high p53 expression together with a high Ki-67 index can predict aggressive pituitary tumor behavior [26, 27]; however, the predictive value of p53 as an independent histopathological marker of aggressiveness has not yet been fully validated, and conflicting results have been reported [28, 29].

Refractory pituitary adenoma is also characterized by rapid growth. However, few reports have examined how to best measure the growth velocity of pituitary adenomas. All of the refractory pituitary adenomas examined here had accelerated growth rates, even when multiple modality treatments had been used. We estimated three-dimensional tumor volumes based on two-dimensional images by multiplying tumor dimensions by slice thickness for each image and summing the volumes of all slices [30]. In a previous study, rapid pituitary adenoma growth was defined as a tumor growth rate greater than 0.07% daily [31]. Because patients with refractory pituitary adenoma are typically examined monthly to monitor tumor progression, we defined rapid growth velocity in refractory pituitary adenomas as greater than 2% monthly. All of the refractory pituitary adenomas examined here grew at a rate greater than 2% per month, indicating that these tumors had malignant features. Thus far, growth rate has not been considered a reliable tool for predicting the clinical behavior of pituitary adenoma, even though it directly reflects important clinical tumor characteristics; we therefore included growth rate as a criterion for the refractory pituitary adenoma classification in order to attach more importance to basic image surveillance.

Another diagnosis criterion for “refractory pituitary adenoma” is early recurrence (<6 months postoperatively), which is also an indicator for malignant pituitary adenomas. After initial surgery, patients with typical adenoma usually experience recurrence after 5–10 years [6, 14]. Refractory pituitary adenomas typically recur even earlier. All of the cases examined here involved early recurrence or tumor regrowth less than 6 months after surgery. This early recurrence (<6 months postoperatively) criterion helps to distinguish refractory pituitary adenoma from aggressive and typical pituitary adenomas, for which time of recurrence is less clearly-defined.

The most important feature of refractory pituitary adenoma is its resistance to conventional treatment and/or salvage therapy with TMZ. This distinguishes refractory pituitary adenoma from aggressive pituitary adenomas, in which response to these treatments is associated with better prognosis. Indeed, “refractory pituitary adenoma” might specifically refer to aggressive pituitary adenomas that are resistant to TMZ and have a poor prognosis, while aggressive pituitary adenomas that do respond to TMZ represent a distinct category. Thus, the TMZ-resistant cases examined here could be considered “refractory pituitary adenomas,” which differ in important ways from more general “aggressive pituitary adenomas.”

The Endocrinology and Neurosurgery departments at the Peking Union Medical College Hospital (PUMCH) are the premier locations for pituitary adenoma treatment in China. Thousands of pituitary adenoma patients visit the PUMCH clinic, and approximately 1000 of them undergo pituitary surgery each year. One third of these patients have invasive, aggressive, atypical, or recurrent pituitary adenomas, which are difficult to manage and are associated with a poor prognosis. Chinese pituitary adenoma patients are not usually diagnosed until the disease has reached a late stage as a result of infrequent examinations or other limitations of the healthcare system. One percent of pituitary adenoma patients in PUMCH are diagnosed with refractory pituitary adenomas. These patients do not always receive appropriate early treatment because some physicians and neurosurgeons are not aware of the severity of these adenomas. We therefore recommend the use of aggressive early treatments, including radiotherapy and/or TMZ, to reduce residual tumor growth in patients with refractory pituitary adenoma characterized by a high Ki-67 index, rapid growth, early and frequent recurrence, and resistance to current treatments. Furthermore, refractory vs. nonrefractory status should be evaluated in addition to invasiveness, typical vs. atypical characteristics, and aggressiveness when radiological findings, pathological features, clinical behavior, and response to treatment are evaluated in pituitary adenoma patients.

In conclusion, the refractory pituitary adenoma classification is crucial for improving patient prognoses; early identification of adenomas with these characteristics might encourage the early use of aggressive therapeutic strategies to prevent, or more effectively delay tumor recurrence.

## SUPPLEMENTARY TABLE



## Abbreviations

ACTH: Adrenocorticotropic Hormone
CT: Computed Tomographic
GKS: gamma knife surgery
H&E: Haematoxylin and eosin
MGMT: O6 methylguanine-DNA methyltransferase
NFPA: non-functional pituitary adenoma
PUMCH: Peking Union Medical College Hospital
PET: Positron-Emission Tomography
RT: radiotherapy
TMZ: temozolomide
TSS: Transsphenoidal surgery
UFC: Urine Free Cortisol
WHO: World Health Organization
