# Supplementary material for: Refractory pituitary adenoma: a novel classification for pituitary tumors
Source: Oncotarget. 2016 Nov 10;7(50):83657–68. doi: 10.18632/oncotarget.13274 (PMC5347795; doi:10.18632/oncotarget.13274)
Supplement: Supplementary file 1 [file oncotarget-07-83657-s001.pdf]

Refractory pituitary adenoma: a novel classification for pituitary tumors

SUPPLEMENTARY TABLE

Supplementary Table S1: Clinical feature of typical pituitary adenomas and pituitary carcinomas

Case	Sex	age (y)	PA	Invasive (Y or N)	Ki-67 (%)	growth rate(%/m)	Atypical (Y or N)	Surgery (n)	RT	medicine	Outcome
1	F	46	NFPA	N	1%	<2%	N	1	N	N	Cured
2	F	31	ACTH	N	2%	<2%	N	1	N	N	Cured
3	F	26	PRL	N	1%	<2%	N	1	N	Y	Cured
4	M	35	GH	N	1%	<2%	N	1	N	Y	Cured
5	F	20	PC	Y	10%	>2%	N	4	GKS and RT	Y	Progression

PC: pituitary carcinoma, GKS: gamma knife surgery; RT: radiotherapy; N: no; Y: yes
